# Point prevalence of appropriate antimicrobial therapy in a Dutch university hospital

**DOI:** 10.1007/s10096-015-2398-6

**Published:** 2015-05-28

**Authors:** H. Akhloufi, R. H. Streefkerk, D. C. Melles, J. E. M. de Steenwinkel, C. A. M. Schurink, R. P. Verkooijen, C. P. van der Hoeven, A. Verbon

**Affiliations:** Department of Medical Microbiology and Infectious Diseases, Erasmus MC, University Medical Center, P.O. Box 2040, 3000 CA Rotterdam, The Netherlands; Department of Internal Medicine, Division of Infectious Diseases, Erasmus MC, University Medical Center, P.O. Box 2040, 3000 CA Rotterdam, The Netherlands

## Abstract

Antimicrobial stewardship teams have been shown to increase appropriate empirical antibiotic therapy and reduce medical errors and costs in targeted populations, but the effect in non-targeted populations is still unclear. The aim of this study was to determine the prevalence of inappropriate antibiotic use in a large university hospital and identify areas in which antimicrobial stewardship will be the most effective. In a point prevalence survey we assessed the appropriateness of antibiotic therapy using an electronic surveillance system in combination with a standardized method for duration of therapy, dosage, dosage interval, route of administration, and choice of antibiotic drug. Patients using at least one antibiotic drug were included. Among 996 patients admitted in the surveyed wards, 337 patients (33.8 %) used one or more antibiotic drugs. Two hundred and twenty-one patients (22.2 %) used antibiotic medication therapeutically, with a total of 307 antibiotic prescriptions. Antibiotic therapy was deemed inappropriate in 90 (29.3 %) of these prescribed antibiotics, with an unjustified prescription as the most common reason for an inappropriate prescription. Use of fluoroquinolones and amoxicillin/clavulanic acid and a presumed diagnosis of fever of unknown origin, urinary tract infection, and respiratory tract infection were associated with inappropriate antibiotic therapy. Our study provides insight into the (in)appropriateness of antibiotic prescriptions in a tertiary care center in the Netherlands and identifies areas for improvement. The use of an electronic surveillance system for this point prevalence study is easy and may serve as a baseline measurement for the future effect of antibiotic stewardship.

## Introduction

Antibiotics are an indispensable part of modern medicine. However, as with all drugs, antibiotics may have adverse effects and medication errors can occur in prescribing. Another untoward effect of antibiotics is the selection of antibiotic-resistant bacteria. In 2007, more than 8,000 excess deaths in Europe were associated with bloodstream infections caused by methicillin-resistant *Staphylococcus aureus* (MRSA) and third-generation cephalosporin-resistant *Escherichia coli* [1]. This mortality is only a fraction of the total burden of disease associated with antibiotic resistance [1]. The U.S. Centers for Disease Control and Prevention (CDC) estimated that each year at least 2 million people in the USA acquire infections with antibiotic-resistant bacteria, with at least 23.000 deaths as a direct result of these infections [2]. Although in the Netherlands antimicrobial resistance is low compared to other countries [3], antimicrobial resistance here is also increasing [4].

A clear relationship has been found between the percentage of resistant strains and antimicrobial use [5]. In addition, only around 60 % of empirically started antibiotics are considered appropriate [6–8]. Finding a balance between adequate antibiotic use for the individual patient, avoidance of selection of antibiotic resistance, and medication errors is the key role of antibiotic stewardship teams (ASTs) [9]. ASTs have been shown to increase appropriate empirical antibiotic therapy and reduce medical errors and costs [5, 10, 11]. Moreover, by narrowing down earlier broad-spectrum treatment, the development of antimicrobial resistance will decrease [5, 11]. In a hospital-wide rollout of antimicrobial stewardship, AST intervention was associated with a large reduction in targeted antimicrobial utilization among patients receiving at least 3 days of antimicrobial therapy, but no significant change was observed hospital-wide [12].

The aim of this study was to determine the prevalence of inappropriate antibiotic use and to identify the areas in which ASTs can have an important impact hospital-wide.

## Materials and methods

### Setting

The Erasmus MC, University Medical Center Rotterdam is a 1,320-bed tertiary care center in Rotterdam with all medical specialties available. In 2012, there were 41,773 admissions and 286,155 bed days.

### Cross-sectional point prevalence survey

The point prevalence survey of antimicrobial use was performed on May 4th and May 16th 2013. Patients were selected with E-Surveillance, an electronic surveillance system, which has been operational in our hospital since 2011 [13, 14]. Originally, it was developed as a tool to automatically select patients suspected of having hospital-acquired infections from a hospital-wide point prevalence population. In this system, patient census data, antibiotic prescriptions, individual antibiotic treatment, infectious disease consultancy reports, laboratory data, microbiological results, vital signs, surgical reports, and radiology reports are integrated. We used E-Surveillance to execute a set of algorithms designed for this study. First, the point prevalence population was automatically created. The study population consisted of all patients in all clinical departments of the Erasmus MC [including a 32-bed general intensive care unit (ICU)], with the exception of the cardiothoracic ICU (18 beds), pediatric (200 beds), and psychiatric wards (77 beds). Then, all patients using at least one antibacterial for systemic use (ATC code starting with J01) on May 4th or May 16th 2013 were marked, with the exception of those patients that received their antibacterial prophylactically. An algorithm differentiated between therapeutic and prophylactic use of the antibacterial based on our hospital’s antibiotic policy. The following antibiotic drugs were defined as prophylaxis and excluded by the E-Surveillance system: cotrimoxazole at a dose of 480 mg, amoxicillin/clavulanic acid given once, and cefazolin started preoperatively, intraoperatively, or in a postoperative period without another clear indication. Antibiotics given regarding a prophylactic protocol, such as selective decontamination of the digestive tract, antibiotics for patients with neutropenia, chronic obstructive pulmonary disease (COPD), and feneticillin within a period of 2 years after splenectomy were also defined as prophylaxis.

### Review of the antibiotic policy

Antibiotic drugs were also considered to be prophylactic if they were recorded as such in patient progress notes. Relevant data elements, such as age, sex, ward, and prescribed antibiotic(s) were retrieved from the E-Surveillance database. The appropriateness of antibiotic therapy was determined for each individual patient by both a clinical microbiologist and an infectious disease consultant, using the standardized method developed by Gyssens et al. [15]. Infection information from the admission day until the prevalence day could be used to assess the appropriateness of antibiotic therapy. Discrepancies were discussed by the reviewers until consensus was reached. Relevant parameters associated with antibiotic use were evaluated and the following classifications were used: appropriate prescription, inappropriate prescription due to incorrect use, incorrect choice or unjustified prescription, and insufficient records for categorization. To evaluate the different relevant parameters a flowchart was used by the reviewers for each prescription, which resulted in classification of the prescription into one of the possible categories shown in Table 1. Antibiotic drug prescriptions could be placed in more than one category if they were inappropriate for more than one reason. All data were reviewed in E-Surveillance and, when indicated, by chart review.

**Table 1:** Categories evaluation of the appropriateness of antimicrobial drug therapy (ADT)

Categories	Absolute frequency	Percentage of total number of prescriptions
I. Appropriate ADT	199	64.8
II. Inappropriate ADT, due to incorrect use:	21^a^	6.8
a. Improper duration	10	3.3
b. Improper route	6	2.0
c. Improper dosage interval	10	3.3
d. Improper dosage	8	2.6
III. Inappropriate ADT, due to an incorrect choice:	25^a^	8.1
a. Allergy to the prescribed antibiotic drug	0	0
b. Less broad-spectrum alternative agent	9	2.9
c. Less expensive alternative agent	7	2.3
d. Less toxic alternative agent	4	1.3
e. More effective alternative agent	15	4.9
IV. Inappropriate ADT, due to unjustified prescription: use of any antimicrobial is not indicated	48	15.6
V. Insufficient information	18	5.9

^a^Four antibiotic prescriptions were inappropriate due to incorrect use and choice

Prescribing therapeutic antibiotics was considered justified for an infection that was either community-acquired or nosocomial. A community-acquired infection was defined as documented or suspected infection within 48 h after admission with fever (>38 °C) and/or elevated infection parameters (C-reactive protein >10 mg/l, white cell count >11 × 10^9^/l, or erythrocyte sedimentation rate >20 mm/h) A nosocomial infection was defined as infection meeting the CDC criteria and occurring at least 48 h after admission.

The definition of appropriateness of antibiotic therapy was based on the current local antimicrobial treatment guidelines, which is in line with the national guidelines (http://www.swabid.nl) and available microbiological results. The antibiotic prescription was defined as inappropriate due to unjustified prescription, when the use of an antibiotic was not indicated because no infection was present. The antibiotic drug prescription was also considered to be inappropriate when the administered antibiotic drug was not in line with the antibiotic guidelines, in case of allergy to the prescribed antibiotic drug, or when a more effective, less toxic, less expensive, and/or less broad-spectrum alternative agent was available. Additionally, antibiotic drug prescription was considered inappropriate in case of incorrect duration, incorrect dosage, incorrect dosage interval, and/or incorrect route of administration (Table 1). For an incorrect dosage, kidney and liver function were taken into account, as well as the (available) antibiotic concentration in blood. The route of antibiotic administration was considered incorrect when a patient was able to switch from intravenous (iv) to oral antibiotic drugs when iv drugs had been given for 48 h, the signs and symptoms of infection had improved, and an oral alternative was available. Criteria that needed to be fulfilled were hemodynamically stable; afebrile (i.e., temperature <38 °C for 24 h); diagnosis and/or pathogen known or highly probable; oral intake possible; absence of factors interfering with drug resorption and/or bioavailability; no contraindications for oral antibiotics; and no significant interaction with other medication.

## Results

### Antibiotic use and demographics

At the start of the survey, a total of 996 patients were admitted on the included wards, of which 337 patients (33.8 %) were using one or more antibiotic drugs. Antibiotic drugs were used prophylactically in 116 patients; these patients were excluded from the analysis. Two hundred and twenty-one patients (22.2 %) used antibiotic medication therapeutically, with a total of 307 antibiotic prescriptions. The median age of patients receiving antibiotic therapy was 62.6 years and 42 % was female. In nearly half of the patients (45.3 %), a clinical microbiologist or infectious diseases specialist was consulted. Twenty patients were admitted on both point prevalence dates. These patients used a total of 64 antibiotic drugs. On both days, 15 of these antibiotics were still prescribed for the same diagnosis. Most patients (68.3 %; 151/221) were treated with one antibiotic drug, 57 patients (25.8 %) were treated with two, and 13 (5.9 %) were treated with three or more antibiotic drugs. Combinations of beta-lactam antibiotics plus or minus beta-lactamase inhibitors were the most commonly prescribed antibiotic class, followed by fluoroquinolones.

### Appropriateness of antibiotic therapy

In total, 90 (29.3 %) of the 307 prescribed antibiotics were classified as inappropriate antimicrobial drug therapy (Table 1). More specifically, for 48 (15.6 %) prescriptions there was no indication for antimicrobial therapy. Twenty-five (8.1 %) prescriptions were an incorrect choice of antibiotic drug, for which a more effective, a less toxic, or a less expensive alternative agent was available. Interestingly, in nearly 36 % of the incorrectly chosen antibiotics, therapy could have been narrowed down. Twenty-one (6.8 %) of the prescribed antibiotic drugs were used incorrectly, mostly due to an incorrect duration of therapy or an improper dosage interval (Table 1).

### Appropriateness of antibiotic therapy according to antibiotic class, diagnosis, and ward

The rate of inappropriate antibiotic therapy varied from nearly 50 % for broad-spectrum antibiotic drugs to 10 % for narrow-spectrum penicillins (Table 2). Antibiotic drugs used as empirical therapy in our hospital, such as amoxicillin/clavulanic acid and second-generation cephalosporins, had a higher rate of inappropriate prescriptions than drugs that are more often used in targeted therapy, such as glycopeptides and third-generation cephalosporins. Antibiotic therapy prescribed for respiratory tract infections (30.6 % of the total prescriptions) was inappropriate in 38 % and for urinary tract infections (20 % of the total prescriptions) in 45 % (Table 3). Most antibiotic drugs were prescribed on only three wards: lung diseases (18.6 %), surgery (17.3 %), and internal medicine (14.7 %). Of all the medical specializations, neurosurgery has the highest percentage of inappropriate antibiotic drug therapy (44 %) (Table 4).

**Table 2:** Appropriateness of antibiotic prescriptions according to the class of antibiotic^a^

Antimicrobial agent	Number of prescriptions (% of total)	Number of inappropriate prescriptions	Proportion of inappropriate prescriptions (95 % confidence interval)	OR^b^ for inappropriate prescriptions (95 % confidence interval)
Fluoroquinolones	37 (12.1)	18	0.49 (0.33–0.64)	Reference
Amoxicillin/clavulanic acid	34 (11.1)	15	0.44 (0.29–0.61)	0.88 (0.34–2.29)
Meropenem	28 (9.1)	5	0.18 (0.08–0.36)	0.24 (0.07–0.77)
Cephalosporins, second generation	27 (8.8)	10	0.37 (0.22–0.56)	0.63 (0.22–1.74)
Piperacillin/tazobactam	26 (8.5)	8	0.31 (0.17–0.50)	0.50 (0.17–1.46)
Glycopeptides	22 (7.2)	1	0.05 (0.01–0.22)	0.05 (0.01–0.39)
Narrow-spectrum penicillin^c^	19 (6.2)	2	0.11 (0.03–0.31)	0.12 (0.02–0.59)
Penicillins with extended spectrum^d^	18 (5.9)	3	0.17 (0.06–0.39)	0.21 (0.05–0.88)
Macrolides	16 (5.2)	6	0.38 (0.18–0.61)	0.75 (0.22–2.60)
Cephalosporins, third generation	15 (4.9)	2	0.13 (0.04–0.38)	0.17 (0.03–0.85)
Metronidazole	12 (3.9)	2	0.17 (0.05–0.45)	0.20 (0.04–1.04)
Aminoglycosides	12 (3.9)	2	0.17 (0.05–0.45)	0.25 (0.05–1.34)
Polymyxins^e^	10 (3.3)	4	0.40 (0.17–0.69)	1.00 (0.22–4.63)
Clindamycin	9 (2.9)	1	0.11 (0.02–0.44)	0.13 (0.01–1.11)
Trimethoprim/sulfonamide	9 (2.9)	6	0.67 (0.35–0.88)	2.00 (0.43–9.26)
Other^f^	13 (4.2)	5	0.38 (0.18–0.64)	0.83 (0.22–3.23)
Total	307 (100)	90		

^a^Eighteen prescriptions could not be assessed because of insufficient information

^b^ *OR* odds ratio

^c^Narrow-spectrum penicillin: penicillin and flucloxacillin

^d^Penicillins with extended spectrum: amoxicillin and piperacillin

^e^Polymyxins: colistin

^f^Other: linezolid, nitrofurantoin, rifampicin, doxycycline, sulfadiazine

**Table 3:** Appropriateness of antibiotic therapy by diagnosis^a^

Diagnosis	Number of prescriptions (% of total)	Number of inappropriate prescriptions	Proportion of inappropriate prescriptions (95 % confidence interval)	OR^b^ for inappropriate prescriptions (95 % confidence interval)
Respiratory tract infection	94 (30.6)	36	0.38 (0.29–0.48)	Reference
Bacteremia	68 (22.1)	11	0.16 (0.09–0.27)	0.33 (0.15–0.72)
Intra-abdominal infection	22 (7.2)	4	0.18 (0.07–0.39)	0.36 (0.11–1.16)
Urinary tract infection	20 (6.5)	9	0.45 (0.26–0.66)	1.53 (0.55–4.22)
Skin and soft tissue infection^c^	15 (4.9)	1	0.07 (0.01–0.30)	0.13 (0.02–1.0)
Fever of unknown origin	13 (4.2)	8	0.69 (0.42–0.87)	3.06 (0.86–10.90)
Other^d^	75 (24.4)	21	0.40 (0.30–0.51)	0.63 (0.33–1.22)
Total	307 (100)	90		

^a^Per antibiotic prescription on date X, 18 prescriptions could not be assessed because of insufficient information

^b^ *OR* odds ratio

^c^Skin and soft tissue infection: erysipelas, cellulitis, hidradenitis suppurativa, panaritium, decubitus

^d^Other: less than ten prescriptions per diagnosis

**Table 4:** Appropriateness of antibiotic therapy by medical specialization^a^

Medical specialization	Number of prescriptions (% of total)	Number of inappropriate prescriptions	Proportion of inappropriate prescriptions (95 % confidence interval)	OR^b^ for inappropriate prescriptions (95 % confidence interval)
Lung diseases	57 (18.6)	16	0.28 (0.18–0.41)	Reference
Surgery	53 (17.3)	17	0.32 (0.21–0.45)	1.25 (0.55–2.82)
Internal medicine	45 (14.7)	12	0.27 (0.16–0.41)	1.03 (0.42–2.48)
Hematology	25 (8.1)	9	0.36 (0.20–0.55)	0.49 (0.15–1.65)
Neurosurgery	18 (5.9)	8	0.44 (0.25–0.66)	2.28 (0.75–6.94)
Gastroenterology/hepatology	16 (5.2)	6	0.38 (0.18–0.61)	1.71 (0.52–5.58)
Neurology	14 (4.6)	6	0.43 (0.21–0.67)	1.92 (0.58–6.42)
Cardiology	14 (4.6)	6	0.43 (0.21–0.67)	2.20 (0.64–7.55)
Urology	12 (3.9)	2	0.17 (0.05–0.45)	0.64 (0.12–3.35)
Orthopedics	10 (3.3)	3	0.30 (0.11–0.60)	1.10 (0.25–4.78)
Thoracic surgery	10 (3.3)	2	0.20 (0.06–0.51)	1.71 (0.26–11.20)
Other^c^	33 (10.7)	8	0.24 (0.13–0.41)	0.98 (0.36–2.65)

^a^ Per antibiotic prescription on date X, 18 prescriptions could not be assessed because of insufficient information

^b^ *OR* odds ratio

^c^ Other: less than ten prescriptions per medical specialization. Medical specialization in this category: ear, nose, and throat; oncology; dermatology; geriatrics; gynecology; radiotherapy

## Discussion

In our tertiary care hospital, antibiotic drugs are used in 33.8 % of the adult patients in general wards and 22.2 % is used therapeutically. Of the patients prescribed antibiotics therapeutically, 90 (29.3 %) antibiotic prescriptions were inappropriate. The highest percentage of inappropriately prescribed antibiotic drugs was due to unjustified use, i.e., no antibiotic use was deemed indicated. Improper dosing intervals and incorrect duration were also commonly found, as well as prescription of an antibiotic drug when a more effective alternative was available. Urinary tract infection and respiratory tract infection were the infections with the highest inappropriate antimicrobial drug therapy. Our data offer areas of possible intervention by antimicrobial stewardship. In the future, repeated audits of the appropriateness of antimicrobial therapy will give insight into the effectiveness of interventions aimed at improving antibiotic drug use and, thus, the effect of ASTs.

Point prevalence surveys are useful tools to assess appropriate antibiotic use [7]. However, the required time investment and limited human resources can constitute a barrier to perform such surveys. The time investment depends on the size of the hospital, the kind of patients, the experience of the reviewer, and a possible combination with other surveys, such as point prevalence studies of infections. The time needed to perform these surveys has been reported to be 10–20 min per patient (personal communication with dr. P.R. Ingram [16] and I. Willemsen [7]). Our study is, to our knowledge, the first to use a computer-based surveillance system to estimate the point prevalence of antibiotic use. With the use of our electronic surveillance system, with automatic selection of patients and extraction of data needed, it took us 5–10 min per patient. Using E-Surveillance, we could determine the prevalence of antibiotic drugs in a shorter time period than other methods, circumventing laborious efforts of the inspection and collection of data on the wards [7, 16].

The prevalence of antibiotic use corresponds to the Dutch point prevalence study in 32 hospitals by the Prevention of Nosocomial Infections through Surveillance (PREZIES) network, which showed that 32 % of all admitted patients (*N* = 9,599) received antibiotic drugs [4]. An Australian hospital-wide point prevalence study showed 47 % inappropriate antibiotic drug use in 199 adult patients from all wards of a tertiary hospital using also the method developed by Gyssens et al. [15, 16]. In contrast to our study, in which risk factors for inappropriate antibiotic prescribing included respiratory infections, fluoroquinolone or amoxicillin/clavulanic acid use, and neurosurgical care, Ingram et al. found bone/joint infections, creatinine level >120 mmol/l, carbapenem or macrolide use, and being under the care of the aged care/rehabilitation team to be risk factors. In a Dutch study 10 years ago in a 1,350-bed teaching hospital including all medical specialties, inappropriate antibiotic use was 37 %, with fluoroquinolone use being the only statistically significant risk factor [7]. The higher inappropriate use in the other two studies may be explained by a difference in time [7], country [16], and the fact that we did not include antibiotic drugs that were given prophylactically. Another explanation might be that in about half of the patients in our study an infectious disease specialist or clinical microbiologist was involved, probably leading to a lower rate of inappropriately prescribed antibiotic therapy [5, 10–12].

Inappropriate use of antibiotic drugs is an important determinant in the development of antimicrobial resistance [17, 18]. For instance, in Europe, antimicrobial resistance is higher in the south of Europe, where much more antibiotic therapy is prescribed compared to Northern Europe [3, 19]. Our study and others [7, 16] have shown that inappropriate use of antibiotic drugs is high, partly because of unjustified antibiotic prescription [20–22]. This may be explained by insecurity about a diagnosis of infection [23], as shown by insufficient documented information for antibiotic use in medical records [24]. Our study provides insight into the areas of inappropriate antibiotic use and, thus, for areas in which interventions may be successful. The importance of the identification of such areas was shown in the rollout of antimicrobial stewardship in a tertiary hospital in Toronto. Among patients meeting stewardship criteria, a 21 % reduction in targeted antibiotic utilization was shown, whereas no significant change was found in all admitted patients [12].

Our study has some limitations. With the electronic surveillance system we used, access to the medical records of patients on cardiac and thoracic ICUs was lacking. Since antibiotic use is high in ICUs, the prevalence of antibiotic use in our hospital may have been lower than expected. However, our results were in concordance with the antibiotic use in other hospitals as shown by the PREZIES data [4]. Another aspect is the inclusion of antibiotics that were prescribed on both days. These antibiotics were included in the analysis because the time difference of 12 days may have resulted in a change of appropriateness of antibiotic therapy, such as, for instance, the duration of therapy.

One of the methods to optimize antibiotic stewardship is a clinical decision support system (CDSS) [9, 25]. Different studies have shown that a CDSS leads to more appropriate antibiotic treatments [26–30]. The surveillance system used in this study has been developed to easily determine infection rates in specialized patient populations, such as postoperative wound infections in surgical patients [13] and will be developed further as an early warning system for nosocomial infections [14]. This system might be upgraded to an integrated computer-assisted decision support system. However, our study has shown that nearly 6 % of patients could not be evaluated for appropriateness of antibiotic use due to insufficient information. This has to be taken into account when a CDSS will be introduced.

In conclusion, our study provides insight into the appropriateness of antibiotic prescriptions in a tertiary care center in the Netherlands and identifies areas for improvement. We used an electronic surveillance system, thereby making the point prevalence study less time consuming and laborious. A point prevalence study for antibiotic use can be an effective tool to assess the effect of antibiotic stewardship either by an AST or a CDSS.
